# Distribution and Morphology of Cortical Terminals in the Cat Thalamus from the Anterior Ectosylvian Sulcus

**DOI:** 10.1038/s41598-019-39327-7

**Published:** 2019-02-28

**Authors:** F. Huppé-Gourgues, R. Abbas Farishta, D. Boire, M. Ptito, C. Casanova

**Affiliations:** 1 École d’optométrie, Université de Montréal, Québec, Canada; 2 Université du Québec à Trois-Rivières, Département d’anatomie, Québec, Canada; 3 Present Address: École de Psychologie, Université de Moncton, Nouveau-Brunswick, Canada

## Abstract

Two main types of cortical terminals have been identified in the cat thalamus. Large (type II) have been proposed to drive the response properties of thalamic cells while smaller (type I) are believed to modulate those properties. Among the cat’s visual cortical areas, the anterior ectosylvian visual area (AEV) is considered as one of the highest areas in the hierarchical organization of the visual system. Whereas the connections from the AEV to the thalamus have been recognized, their nature (type I or II) is presently not known. In this study, we assessed and compared the relative contribution of type I and type II inputs to thalamic nuclei originating from the AEV. The anterograde tracer BDA was injected in the AEV of five animals. Results show that (1) both type I and II terminals from AEV are present in the Lateral Posterior- Pulvinar complex, the lateral median suprageniculate complex and the medial and dorsal geniculate nuclei (2) type I terminals significantly outnumber the type II terminals in almost all nuclei studied. Our results indicate that neurons in the AEV are more likely to modulate response properties in the thalamus rather than to determine basic organization of receptive fields of thalamic cells.

## Introduction

The thalamus has classically been viewed as a necessary relay for the transfer of sensory information from the periphery to the neocortex where complex computations take place along numerous cortical areas organized in a hierarchical manner. In the visual system, this cortico-centric view suggests that most, if not all, neuronal processing leading to perception and action is based on computations within and across cortical areas through direct cortico-cortical connections^1,2^. This view has been challenged by a growing number of studies showing that all visual cortical areas are reciprocally connected to the main extrageniculate thalamic nucleus, the pulvinar, providing then monosynaptic trans-thalamic pathways between areas of the neocortex^3,4^.

Studies from our laboratory and others have shown that neurons in the pulvinar contribute to the processing of basic and complex visual information^5–7^ and there are assumptions that trans-thalamic pathways can be used to facilitate the cortical flow of information^8^ and regulate cortical activity according to attentional demand^9^. Still, the role of the cortical pathways involving the pulvinar remains to be clearly determined since there is little information about the function of the distinct trans-thalamic pathways and how they differ from their corresponding direct cortical-cortical pathways^10^.

One way towards a better understanding of these pulvinar-cortical routes is to determine the nature of the signals they convey. In this context, two types of corticothalamic (CT) axons have been identified based on their morphology^11,12^. Type I axons are thin and possess long thin branches with occasional *en passant* swellings and “drumstick-like” side branches with small terminal endings^13^ considered to be equivalent to the round small (RS) presynaptic terminals observed at the ultrastructural level^14,15^. These small axons arise from layer VI cortical neurons^16,17^. Type II axons are characterized by large caliber that bear clustered endings considered equivalent to the round large (RL) presynaptic terminals^14,15^. These large caliber axons arise from layer V cortical neurons^17,18^. The grouping of their terminals ranges between lone singletons and more complex flowery forms of rosettes^19^ and are often grouped in complex arrangements, many encapsulated within complex glomeruli, and make synaptic contact with multiple profiles^20–22^. Importantly, different functional roles have been attributed to type I and II terminals. According to the ‘driver/modulator’ theory of glutamatergic pathways involving the thalamus and the cortex, type II terminals would be the main carrier of sensory information (driver inputs), while type I would fine-tune ongoing activity (modulatory inputs)^23^.

Several neuroanatomical studies revealed that the proportion of type I and type II cortico-pulvinar terminals vary according to the cortical areas. For instance, in the cat, the vast majority of axons coming from the primary visual cortex have type II terminals while those from the posteromedial lateral cortex (PMLS, an extrastriate area considered as the homologue of the primate area MT) exhibit type I terminals^15^. These results suggest that the nature of the cortical projections to extrageniculate thalamic nuclei varies as a function of cortical hierarchy, characterized by an increase of the modulatory/driver inputs ratio.

In this study, we investigated the morphology and distribution of axon terminals originating from the anterior ectosylvian visual area (AEV), an associative area located in the anterior ecstoylvian sulcus (AES) considered as one of the highest areas along the hierarchical organization of the cat visual system^24^. The AES comprises three modality-specific subregions: the somatosensory SIV^25^, the auditory FAES^26^, and the visual AEV^27^. Multisensory neurons are present in each subregion but are mainly situated at their common borders^28–30^. The AES is also related to the motor and limbic systems^31^, suggesting that it might play a role in the animal’s orientation and alerting behavior^32^. Injections of an anterograde tracer were made in visual and multisensory cortical subregions of the AES and terminals reaching the main thalamic targets were characterized, i.e. in the tecto-recipient zone the pulvinar complex (namely, the medial lateral posterior nucleus (LPm), the lateral median suprageniculate complex (LM-Sg), the medial subdivision of medial geniculate (MGm)^27,33–35^, and the posterior nucleus group (PO)^33^. Our results indicate that most CT axons are type I terminals, supporting the assumption that the proportion of driver/modulator inputs vary along the cortical hierarchy.

## Materials and Methods

Animals were treated in accordance to the regulations of the Canadian Council for the Protection of Animals (CCPA). The protocols were approved by the ‘Comité de déontologie de l’expérimentation sur les animaux’ of the Université de Montréal. Five pigmented adult cats were used in this study. Pre-operative anti-inflammatory agents (tolfedine 4% s.c. 0.1 mg/kg) and antibiotics (tribrissen 24% s.c. 0.13 ml/kg) were administered 24 hours before surgical procedures. Twenty minutes before surgery, atropine (0.1 mg/kg s.c.) and Atravet (0.05 mg/kg s.c.) were given to the animal. Anesthesia was induced with a mixture of 5% Isoflurane in 60% N_2_O and 25% O_2_ and maintained with 2% Isoflurane in the same gaseous mixture of N_2_O and O_2_. Animals were positioned in a stereotaxic apparatus. A craniotomy was performed over the injection sites. During all surgical procedures, animals were maintained at 38 °C and heart rate, end-tidal CO_2_, blood O_2_ saturation and blood pressure were closely monitored.

AES craniotomies were performed according to Horsley-Clarke (H-C) coordinates from 10 to 15 mm lateral to the midline and 10 to 15 mm anterior to the interaural plane. Injections were made under electrophysiological monitoring to ensure the position of the pipette into the cortex. Borosilicate pipettes (1.5 mm external diameter) were pulled to obtain a tip ranging between 20 and 30 µm. Biotinylated dextran amines (BDA 3000kD) were injected (Midgard, Stoelting) in AES by iontophoresis using DC current (7 second on/off cycle 7 µA) for 20 minutes. Cases in which the injections encroached the white matter were rejected (one case, AES 6 had minimal encroachment and was retained in the analysis). In order to label CT cells of the AES, WGA-HRP (3 µl) was injected in the thalamus with a Hamilton syringe in one animal. Craniotomies were sealed with acrylic bone cement and the wounds were sutured in anatomical layers. Anti-inflammatory and antibiotic treatments were administered pre- and post-operatively and analgesic was applied for 48 h following surgery (temgesic 0.01 mg/kg bid).

**Figure 1:**
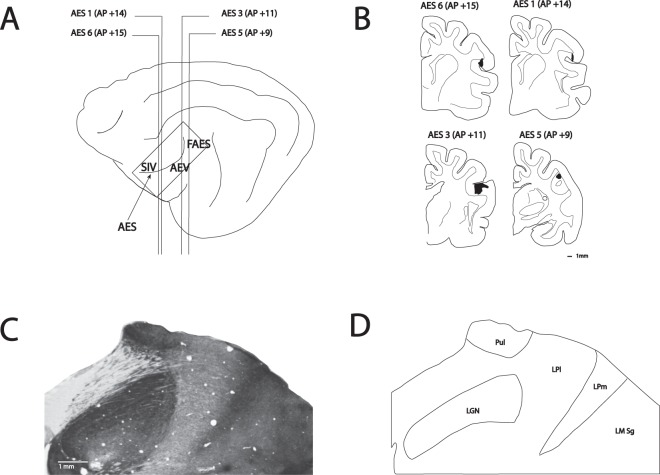
(**A**) Representation of the brain showing the location of the subregions of the AES cortex. (**B**) Cortical injection sites of the illustrated cases. (**C**,**D**) Example of a coronal section stained with AChE used to identify thalamic subregions.

Seven to ten days after the cortical injections, animals received an overdose of sodium pentobarbital (80 mg/kg; IP) and were perfused with phosphate buffered 0.9% saline (PBS: 0.1 M, pH 7.4) followed by phosphate buffered 4% paraformaldehyde. Brains were blocked stereotaxically, removed from the cranium, post fixed overnight in the same fixation solution at 4 °C, cryoprotected in 30% sucrose in 0.1 M phosphate buffer (pH 7.4) and frozen until processed. The fixed brains were cut into 50 µm-thick coronal sections using a cryostat and collected in PBS. After pre-incubation in 2.5% bovine serum albumin (BSA) and normal goat serum 2% in phosphate-buffered saline (PBS; 0.01 M PB with 0.9% NaCl, pH 7.4) for 30 min, BDA was visualized with avidin-biotin-peroxidase complex (ABC; Vectastain ABC Elite kit; Vector, Burlingame, CA). Following buffer washes, sections were reacted with nickel-intensified diaminobenzidine (DAB)^36^ for 10 min. After PBS washes, sections were mounted on slides, dehydrated, mounted with Depex and coverslipped for light level examination. Adjacent sections were processed for acetylcholinesterase histochemistry for the identification of cytoarchitectonic boundaries between the lateral and medial subdivisions of the lateral posterior thalamus^37^ (Fig. 1). Sections were incubated for six hours in an aqueous solution with 50 mM sodium acetate, 4 mM copper sulfate, 16 mM glycine, 4 mM *S*-acetylthiocholine and 86 µM ethopropazine adjusted to pH 5. Sections were rinsed in water and reacted for 10 min in a 1% aqueous solution of sodium sulfite and subsequently fixed in 4% paraformaldehyde for 2 h.

The animal that received a thalamic injection of WGA-HRP was perfused with a short rinse of phosphate buffer (PB) 0.1 M followed by 1% paraformaldehyde 3% glutaraldehyde in PB. Brains were cryoprotected in buffered 30% sucrose. HRP-TMB histochemistry was performed according to the method of Mesulam *et al*.^38^.

For stereological analysis, evenly spaced sections were randomly selected. The sampling was performed according to a systematic random sampling scheme^39^. Drawings of the sections were made with a 10× objective on a microscope (DMR, Leica) linked to a motorized computer-controlled stage and with a reconstruction software (Novaprime, Bioquant). Mapping of the distribution of axon terminals was carried out under a 100×, 1.25 PH3 oil immersion lens. Unbiased size distribution of terminal boutons was obtained using an optical fractionators sampling scheme^40,41^. Briefly, the entire region containing axonal terminals was systematically sampled with optical dissectors ranging between 400 and 2500 µm^2^ in area and 10 µm thick. Sampled area varied from 8% to 100% of the terminal fields of the selected sections. The total number of terminals is presented for every case and every nucleus (see Table 1). Care was taken to avoid sampling in the 2–3 µm immediately adjacent to the sections surfaces to avoid measuring cut or damaged terminals. For comparison purpose, previous cases from PMLS cortex and area 17^15^

**Table 1:** Stereological sampling data (number of counted terminals and percentage of tissue sampled) of cases by nuclei. FO: First order thalamic nuclei, HO: Higher order thalamic nuclei.

	FO				HO					
	MGM	MIN	LGNd	LGNv	MGd	LMSg	PO	LPm	LPl	LPI2
AES1						71 (100%)	81 (100%)			
AES2						8 (100%)				
AES3	25 (27%)				439 (24%)	38 (25%)		84 (23%)		
AES5	191 (12%)				358 (11%)	36 (100%)	76 (2%)	24 (29%)		
AES6	64 100%				69 (5%)	938 (100%)		10 (100%)		
PMLS1				19 (25%)				384 (23%)	140 (15%)	
PMLS2		759 (4%)						42 (1%)	566 (1%)	
PMLS3		26 100%						60 (24%)	32 (25%)	
PMLS4									121 (8%)	36 (24%)
PMLS5									112 (17%)	10 (20%)
A17		166 (8%)							322 (26%)	

were reanalyzed.

The criteria used for the morphological classification of the axon terminals were those of Guillery *et al*.^19^. Briefly, we identified type I as small caliber axons with sparse beaded terminals linked by a small stalk (drumstick). Type I axon terminals also included small swellings on fine caliber axons. Type II axons were identified as (1) intermediate, i.e., comprising three terminals swellings in a small cluster; (2) rosette-like terminals, i.e., complex cluster of more than 3 terminals; and singletons, i.e., single beaded axon endings^42^.

The proportion of each terminal type between the sampled thalamic nuclei was compared with the G-test^43^, while the percentage of each type of terminals arising from cortices located in the AES and PMLS cortex was compared with a Mann-Withney U test. Finally, Kendall’s Tau rank correlations were computed, with the inclusion of area 17 data to further investigate the possible relationship between the cortical hierarchical order and the axon terminal types. All data presented here are available upon request.

## Results

### Injection sites in AES

Injections in the cortices within the AES were made at various locations to target its different sensory sub-regions^27^. In case AES1, the injection site was small and located in the dorsal bank of the rostral part of the AES (H-C +14) in somatosensory and visual regions^29,30^. In case AES2 (not illustrated), the injection was also small and located in the dorsal bank of the caudal end of the ectosylvian gyrus. The injection in case AES3 was located in the ventral bank of the caudal ectosylvian gyrus (H-C +11) in a visual region. In case AES5, the injection was made in the dorso-caudal bank of the ectosylvian gyrus (H-C +9) in visual and auditory regions. Finally, for case AES6, a large injection was made in the dorsal bank of the rostral ectosylvian sulcus (H-C +15) in somatosensory and visual regions. Injection sitelocations and coordinates are shown in Fig. 1.

### Terminals Labeling Following AES Injections

#### General observations

Table 2

**Table 2:** Percentage of thalamic terminal types according to their cortical origin.

%	FO				HO				
	MGM	MIN	LGNd	LGNv	MGd	LMSg	PO	LPm	LPl
**AES1**									
Type1						93,4	98,8		
Inter/Rosette						0,0	0,0		
Singleton						6,6	1,2		
**AES2**									
Type1						100,0			
Inter/Rosette						0,0			
Singleton						0,0			
**AES3**									
Type1	67,9				83,4	89,9		100,0	
Inter/Rosette	10,5				13,8	7,6		0,0	
Singleton	21,6				2,8	2,5		0,0	
**AES5**									
Type1	100,0				93.5	21,7	99,8	71,7	
Inter/Rosette	0,0				1.73	56,5	0,0	23,6	
Singleton	0,0				4.96	21,7	0,2	4,7	
**AES6**									
Type1	100,0				95,9	100,0		100,0	
Inter/Rosette	0,0				4,1	0,0		0,0	
Singleton	0,0				0,0	0,0		0,0	
**PMLS1**									
Type1				36,8				61,2	67,9
Inter/Rosette				36,8				20,8	22,1
Singleton				26,3				18,0	10,0
**PMLS2**									
Type1		98,9						99,7	99,4
Inter/Rosette		0,8						0,3	0,5
Singleton		0,2						0,0	0,1
**PMLS3**									
Type1		100,0						54,7	81,8
Inter/Rosette		0,0						7,2	0,0
Singleton		0,0						38,1	18,2
**PMLS4**									
Type1									74,4
Inter/Rosette									1,8
Singleton									23,8
**PMLS5**									
Type1									31,8
Inter/Rosette									26,3
Singleton									41,9
**A17**									
Type1		100,0							10,9
Inter/Rosette		0,0							28,3
Singleton		0,0							60,9

The stereologic samplings of thalamus terminal fields were calculated from injections in area17, PMLS and AES. This quantification of the different types of terminals permits comparisons according to cortical origin and thalamic localization of corticothalamic terminals. PMLS cases are from a previous study.

shows that all AES injections resulted in labeling numerous projections in the thalamus, including the LPm of the LP ‐ Pulvinar complex.

Given its multisensory function, several other thalamic nuclei were labeled besides the pulvinar. Terminals in the MGm and MGd, first and higher order nuclei of the auditory thalamus respectively, were shown to receive almost exclusively type I terminals (AES 3, 5 and 6). Higher order thalamic nuclei such as the LM-Sg were also predominantly marked by type I terminals (except AES 5). In two cases, injections in the AES labeled cells in the PO and all projections were exclusively type I (99%). Finally, in line with our initial hypothesis, projections to the LPm subdivision of the pulvinar complex were also shown to be exclusively type I in two cases and predominantly type I in one case.

#### Illustrated cases

**Figure 2:**
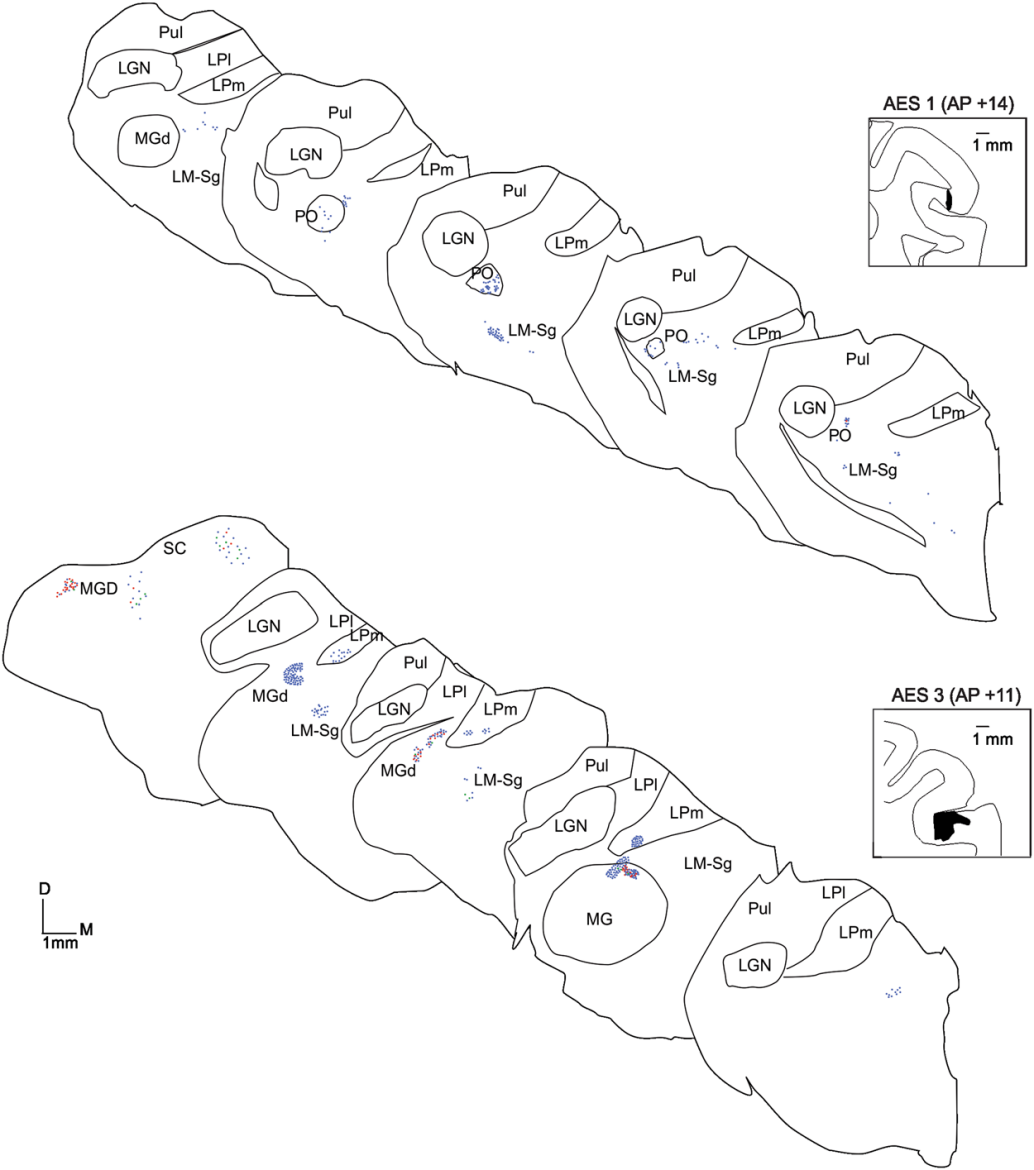
Topographical representation of axon terminals in the thalamus after injection of AES. Injection sites are presented in insets. Blue dots: type I axon terminals, Green dots: Singletons, Red dots: type II axon terminals. In Figs 1A and **2B**, one dot represent one terminal. In Figs 1B and **2A** one dot represents 5 terminals. Scale 1 mm.

**Figure 3:**
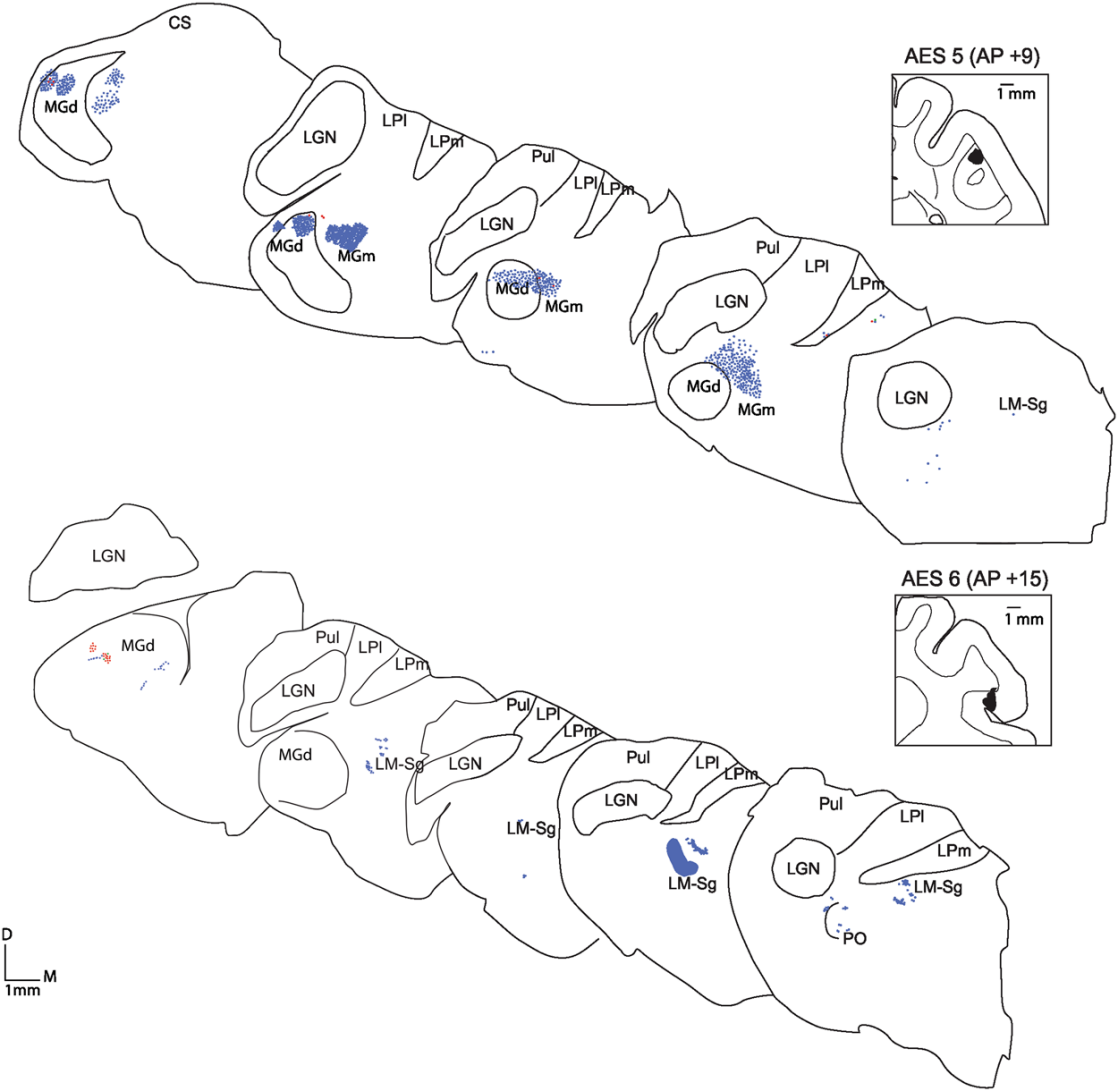
Topographical representation of axon terminals in the thalamus after injection of AES. Injection sites are presented in insets. Blue dots: type I axon terminals, Green dots: Singletons, Red dots: type II axon terminals. In Figs 1A and 2B, one dot represent one terminal. In Figs 1B and 2A one dot represents 5 terminals. Scale 1 mm.

The predominance of type I (blue dots) over type II (green and red dots) terminals across the thalamus can be better appreciated in Figs 2 and 3 where the topographical location of CT projections from four cases are represented with respect to their morphology. In all cases, nuclei, and topographical locations, labeled CT projections were either exclusively or predominantly type I.

In case AES1 (Fig. 2A), the terminals were observed in the LM-Sg, in its ventrolateral tip near the border with the LPm. Some retrogradely labeled cells were also observed therein suggesting a reciprocal thalamo-cortical (TC) projection to the AES. More rostrally, terminals were also found in LM-Sg and PO. In both thalamic regions, axon endings were almost exclusively type I terminals. The proportion of terminals in LM-Sg and PO was not significantly different (Chi-square, p = 0.08).

**Figure 4:**
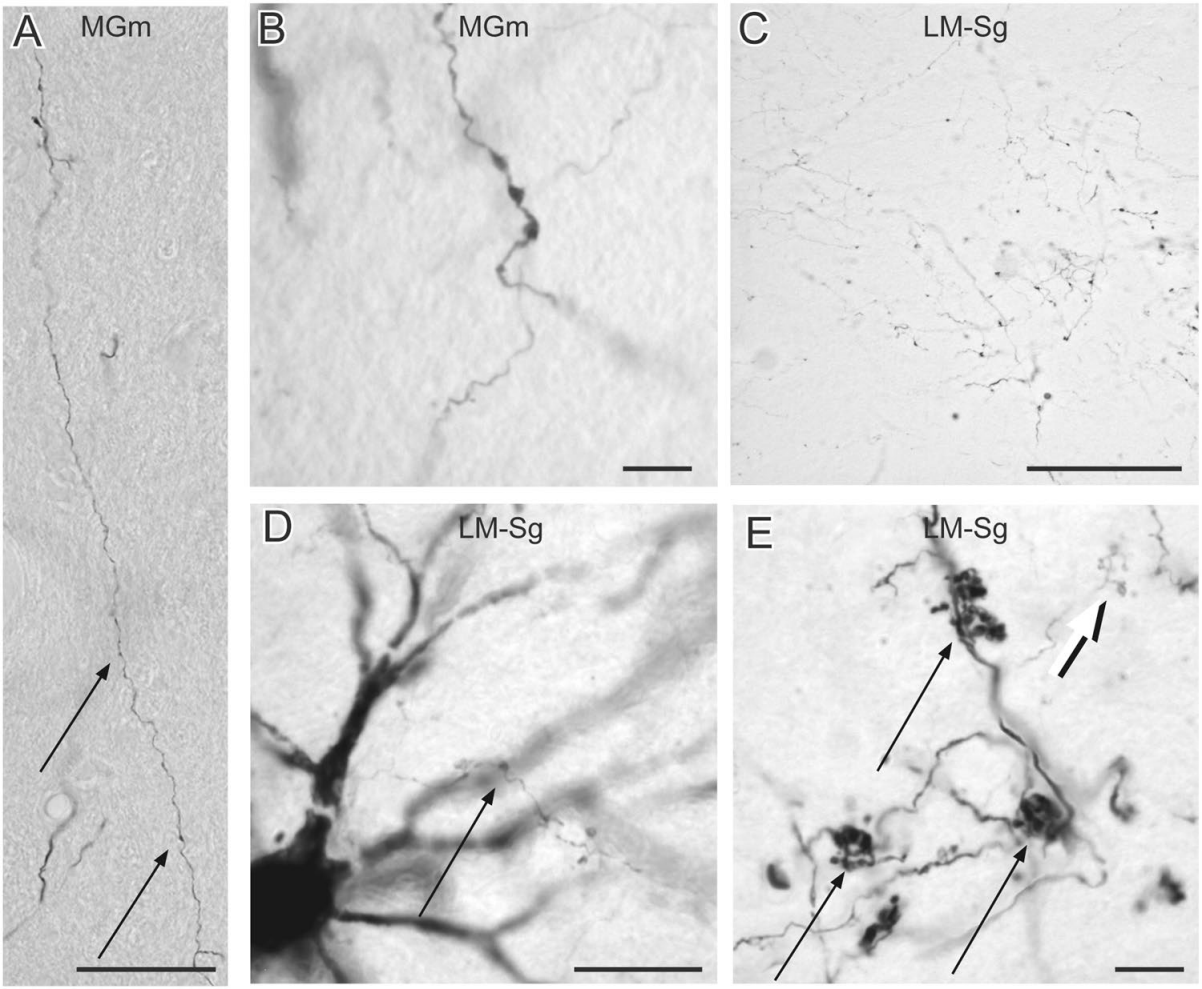
Photomicrograph of corticothalamic terminals. (**A**) Reconstruction of a typical type I axon bearing small sparse terminals (indicated by arrows) found in the MGm. (**B**) Example of en-passant boutons along a corticothalamic axon of the MGm. (**C**) Typical terminal field containing mostly type I in the LM-Sg. (**D**) Type I axon terminals in the dendritic field of a thalamocortical cell in LM-Sg. (**E**) Example of a large cluster of type II axon terminals (black arrow). For comparison, note the size of type I axon passing in the background (white arrow). Scales: A: 50 µm; B, D and E: 10 µm; C: 100 µm.

**Figure 5:**
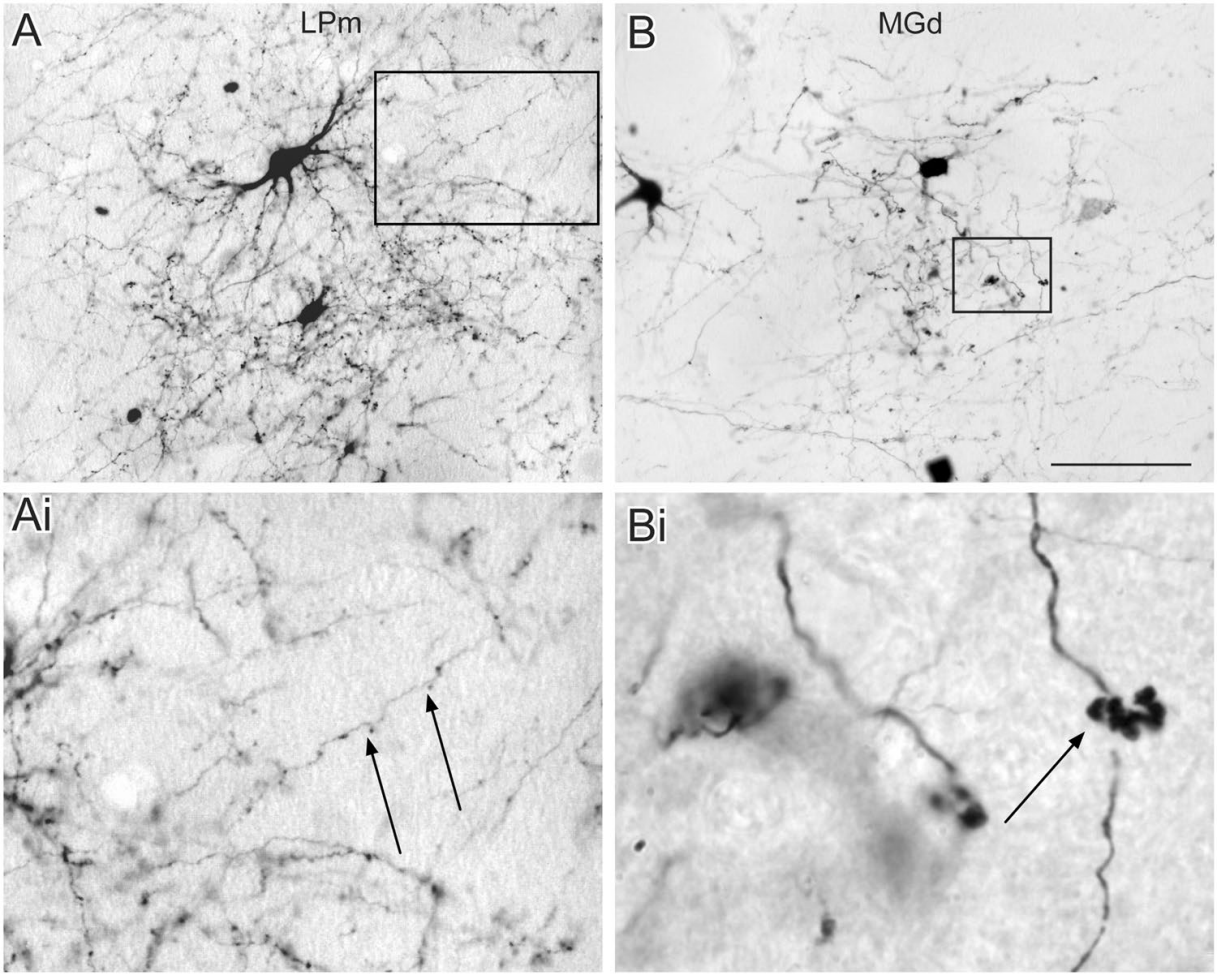
Example of terminal fields found in the thalamus following injection in the AES (from case No 3). (**A**) A focus containing mostly type I axon terminal in the LPm thalamic region. (**B**) A focus containing both type I and II axon terminals in the MGd. Scale in panels A and B is 100 µm.

Panel B of Fig. 2 shows case AES3. In this animal, type I projections were also predominantly found in the first-order nucleus, the MGm, and in three higher-order nuclei, namely, LM-Sg, MGd and LPm. In the MGm nucleus, most axon terminals were type I and characterized by en-passant boutons (Panels A and B in Fig. 4). Foci of both type I and II terminals were seldom observed in LM-Sg (Panel C). Type I terminals were occasionally found coursing within the dendritic arborization of retrogradely labeled cells in LM-Sg (Panel D). In rare occasions, complex structures corresponding to tight aggregates of more than 20 type II terminals were observed (Panel E). Fig. 5 shows axons endings in LPm nuclei of the pulvinar complex and MGd in case AES3. Terminal fields in LPm contained almost exclusively type I axon terminals (Panel A and Ai of Fig. 5) while they were predominantly type I for the MGd (panel B and Bi). The proportion of type I and II terminals were significantly different in the labeled thalamic nuclei of each case (G = 178, df = 6, p < 0.01). Injection of the AES in case 3 also yielded the labeling of type I -like and singleton-like terminals in the *stratum griseum intermedium* of the superior colliculus.

Panel A of Fig. 3 shows case AES5 where only type I terminals were found in the MGm nucleus. Type I terminals largely predominated in PO whereas type II prevailed in LM-Sg (G = 267.9, df = 6, p < 0.05). This case contrasts with AES1 in which type I terminals predominated in both PO and LM-Sg. Both type I and type II terminals were encountered in the LPm, the former being more prominent. Retrogradely labeled cells were observed in the PO nucleus.

In case AES6 (panel B of Fig. 3), only type I terminals were observed in MGm, in close proximity to retrogradely labeled cells. Similarly, only type I endings were found in LM-Sg and LPm nuclei. The MGd nucleus contained a few type II terminals and differed significantly from the other labeled nuclei (G = 53.5, df = 6, p < 0.01). Retrogradely labeled neurons were found in MGm, LM-Sg and PO. A small group of type I-like terminals was observed in the *stratum griseum intermediale* of the superior colliculus.

### Terminals Labeling Following area 17 and PMLS Injections

#### Area 17

Injection of BDA in area 17 resulted in terminal labeling in the LGNd and lateral subdivision of the LP nuclei (LPl) of the pulvinar (Table 2). In the LGNd, only type I terminals were found, while a majority of type II singletons were found in LPl (60.9%). The proportion of terminal types in LGNd and LPl were significantly different (p < 0.01).

#### PMLS cortex

Injections in various PMLS locations resulted in terminal labeling in first-order thalamic nuclei: the MIN in cases PMLS2 and PMLS3 and the LGNv in case PMLS1 (Table 2). Terminals of the cortical fibers reaching the MIN were almost exclusively type I terminals. In all five animals, axon terminals were observed in the LP nucleus: in the LPl and LPm subregions for PMLS 1, 2 and 3 and in the LPl only for the remaining two cases. In both LPl and LPm, terminals were mostly type I, with the exception of case PMLS5 in which type II terminals prevailed. In all cases, the relative frequency of terminal types was always different between first-order and higher-order nuclei. In case PMLS1, the relative frequency of terminal types was significantly different between the LGNv, LPm and LPl (G = 53.4, df = 4, p < 0.01). The proportion of terminal types in LPl and LPm was also significantly different (G = 31.8 df = 4, p < 0.01). In case PMLS2, the relative frequency of terminal types between the MIN and the LPl and LPm is significantly different (G = 50.6, df = 4, p < 0.01). While terminal types in LPm and LPl nuclei did not differ (G = 2.86, df = 4, p > 0.05), endings in each of the LP subdivisions were significantly different from those found the MIN (G = 36.53 and 27.6, df = 4; p < 0.01). In case PMLS3, the percentage of terminal types was significantly different in the MIN, LPL, and LPm (G = 27.75, df = 4, p < 0.01) and the proportion of terminals in LPl and LPm also differed significantly (G = 25.8, df = 4, p < 0.01). Cases PMLS4 and 5 resulted in terminal labeling in the LPl nucleus only.

### Overall Comparison Between Areas

In first-order thalamic nuclei, the percentage of each type of terminals arising from AES and PMLS cortex was not significantly different (Mann-Withney U, p > 0.05). Injections in these cortices resulted mainly in the labeling of type I terminals. In higher-order thalamic nuclei, the mean percentage of type I terminals from the AES (87.88%) and PMLS (71.36%) was significantly different (Mann-Whitney U, p = 0.044). This difference is enhanced if the cases in which the proportion of type I was the lowest (AES5 and PMLS 5) are held from the analysis (Mann-Whitney U, p = 0.026). AES injections resulted in a mean percentage of type II singletons (3.31%) inferior to that from PMLS injections (18.76%) (Mann-Withney U, p = 0.026). In other words, these results indicate that cortico-thalamic neurons from the AES comprise predominantly type I terminals.

**Figure 6:**
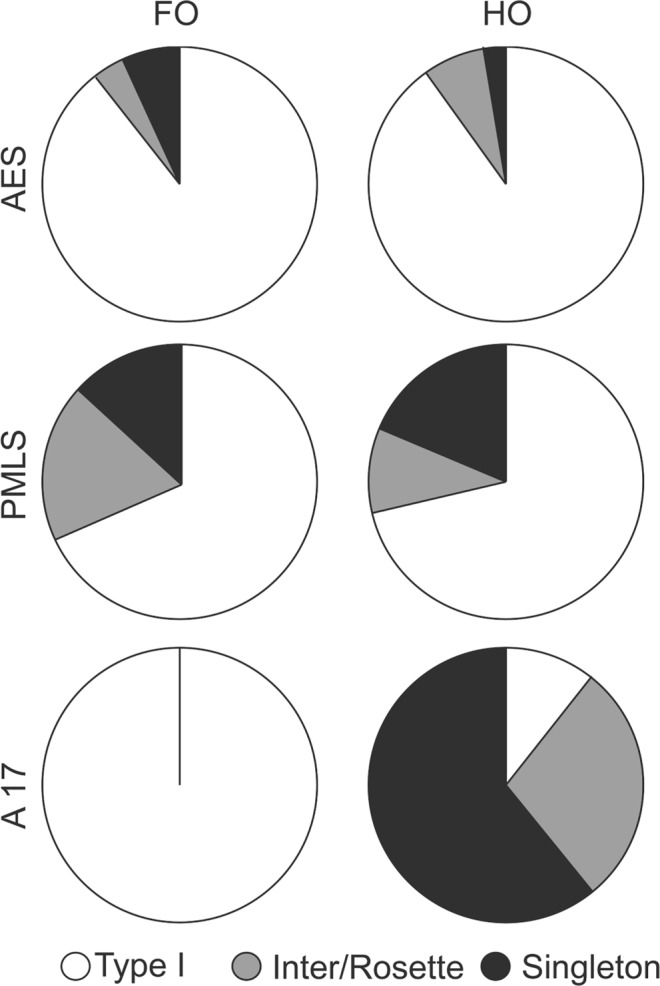
Proportion (%) of terminal types according to their cortical origin and thalamic targets.

Kendall’s Tau rank correlations were computed to further investigate the possible relationship between the cortical hierarchical order and the axon terminal types with the inclusion of area 17 data. For first-order thalamic nuclei, there is no significant correlations between the cortical areas (and consequently, their hierarchical order) and the relative frequency of terminal types. For higher-order thalamic nuclei, a highly significant positive correlation was found between the cortical hierarchical order and the percentage of type I terminals (Tau = 0.468, p = 0.012). In addition, a very significant negative correlation was found between the hierarchical order and the percentage of singleton terminals (Tau = −0.568, p = 0.006) (Fig. 6). That is to say that the number of type I terminals increases while the number of singletons (type II) decreases with increasing hierarchical levels of visual cortical area (from area 17 to PMLS to AEV).

### Retrograde Labeling

**Figure 7:**
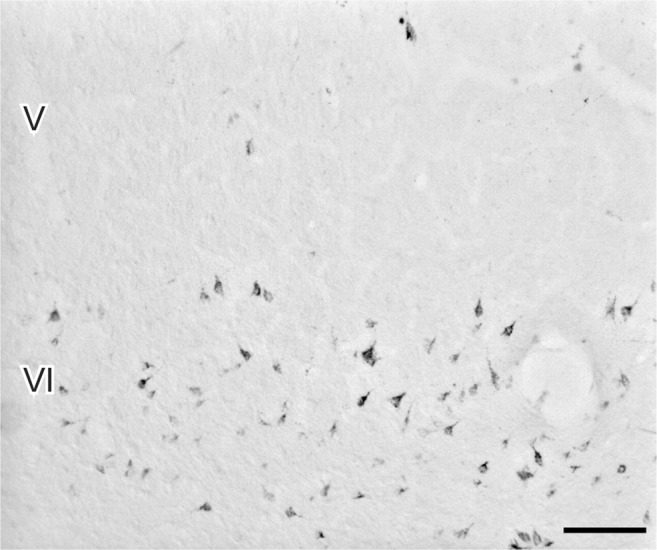
A large injection of WGA-HRP in the thalamus reveals retrograde labeled cells in the AES. These neurons were mainly found in layer VI. Scale 100 µm.

Corticothalamic cells in the AES were visualized following retrograde transport of WGA-HRP injected in the medial thalamus comprising the LPm, LM-Sg, and MG nuclei. Retrogradely labeled cells were predominantly found in layer VI (with rare cells in layer V) as shown in Fig. 7.

## Discussion

The present results indicate that most CT axons of the visual and multisensory subregions located in the AES targeting higher-order nuclei exhibit type I terminals. This is in contrast with area 17 which provides mostly type II terminals (including intermediate, rosettes, and singletons) and PMLS cortex which presents a combination of all terminal types. Thus, these results are in agreement with the initial assumption that the proportion of type I versus type II axon terminals in higher order thalamic nuclei increases with the hierarchical order of cortical visual areas.

Most of the tracer injections were small in order to avoid contamination of the white matter underlying the AES. The injection and projection sites correspond to the electrophysiological distribution of sensory specific territories^27,44–46^. Our injections were mainly aimed at the visual portion of the AES as confirmed by the presence of terminals in visual thalamic nuclei such as the LPm and LM-Sg. However, injections were not restricted to a single sensory modality. In cases AES5 and AES6, injections were made in the caudal part of the AES where auditory and/or visual responsive cells are present^27,47^. The most caudal injection in AES5 resulted in more labeled terminals in the MG than any other injection sites^48^. These results are in agreement with previous tracing studies^27,31,33,34^.

First order thalamic nuclei receive their driving afferents from ascending sensory pathways and transmit information to the cortex. They also receive type I axon terminals from layer VI cells of their respective primary sensory cortices^17,49^. MGv and LGNd are the first order thalamic relays of the auditory and visual system respectively^50^ for, the, review. Guillery (1995) has defined MGm as a first order auditory relay since it receives its sensory driving input form from the inferior colliculus^51^. Our results show that MGm receives both type I and II terminals. This contrasts with the visual system organization as the LGNd does not receive direct projections from AES. The difference between these two modalities may be explained by a different phylogenetic origin of these two nuclei^52^, b^53^ and may represent specificities of the two modalities.

It is generally considered that higher-order thalamic nuclei receive their driving afferents from layer V of the cortex and modulatory signals from layer VI^17,22,49^. Our results show that projections from the AES to the MGd, PO and the medial part of the pulvinar complex are predominately type I and originate almost exclusively from layer VI. In one case only (AES 5), there was a greater number of type II terminals in the LSMG (Table 2), but still in minute quantity. The other nuclei of the same animal exhibited a profile comparable to all other cases (i.e. a higher number of type 1), leaving only the LMSG of AES5 to stand out from the others. AES5 was the most posterior injection of all made in the cortex. Nervertheless, the vast majority of the thalamic nuclei investigated here (12 out of 13) received type I projections suggesting that the cortical areas from the AES exert a modulatory influence on these higher-order thalamic nuclei. This is in contrast with the predominance of a driver input from the primary visual cortex on higher-order visual nuclei.

Our data also highlights the fact that the proportion of type I over type II terminals may increase along the hierarchical order of visual areas. If that is correct, the CT projections from the primary visual cortex would be the main source of driving inputs to the pulvinar while higher-order cortical areas are mainly involved in the fine-tuning of its ongoing activity. This modulatory action would be more and more prominent as one goes from lower to higher-order cortex. These results are in agreement with functional studies showing a significant decrease of receptive field responsiveness of a number of cells in the cat LPl^54,55^ and in the inferior pulvinar of primates^56^, following inactivation of the primary visual cortex.

The LM-Sg, one of the main targets of the subregions from the AES, is involved in multisensory information processing^57,58^. Our results show that both types of axon terminals are located in LM-Sg but that the modulatory type I largely predominates the corticothalamic projection from the AES. This contrasts with its axons originating from cells in the superior colliculus that have type II terminals and thus exert a driver influence^31,59^. It is not known whether the corticothalamic pathway conveys multisensory information to LM-Sg. In view of the fact that LM-Sg receives a significant input from intermediate layers of the superior colliculus and that terminals are similar to type II and retinal RLP terminals, it could be proposed that multisensory properties of LM-Sg cells are provided by the superior colliculus input and that LM-Sg responses can be further modulated by AES layer VI cell signals.

The observation of terminals in close proximity to dendrites of retrogradely labeled cells raises the possibility that cortices locate in the AES participate in corticothalamocortical loops. These loops can be either strictly reciprocal loops or feedback pathways to other early visual areas^24^. Both assumptions are supported by the results of Miceli *et al*.^60^ who reported that a group of neurons in the LPm/LM-Sg region send collaterals to both PLLS and AES cortices. Whether cortices in the AES entertain particular reciprocal connection with such divergent relay cells is presently unknown. However, these neurons were situated in similar locations and it may be possible that the cortical areas in the AES modulate their gating properties.

To our knowledge, only a few studies have investigated the proportion of type I/type II CT terminals in the pulvinar across species, especially for cortical areas beyond the primary visual cortex. Most data about the morphology of CT terminals in the pulvinar come from the cat model. In this animal, we presented evidence that the projections from area 17 to the LPl are mainly characterized by type II terminals (driver input)^15^, while axon terminals from the PMLS cortex are both type I and II. Cortico-thalamic projections from areas 18, 19, 20a and b, 21a, 5 and 7, and AMLS cortex have been reported in the LP pulvinar complex by several authors^37,61–63^, but none of these studies specifically investigated the driver/modulator nature of these projections.

As far as we know, studies in primates were limited to V1 and area MT. As revealed in cats, axon terminals from the primary visual cortex to the pulvinar exhibit a type II like morphology, while projections from MT are predominantly characterized by type I terminals^12,64^. However, since these studies were done in the context of axon and arbor reconstruction, the exact proportion of type I/type II is unknown. We are not aware of any other studies in primates that systematically quantified the number of type I and II boutons of corticopulvinar axon terminals. Therefore, it remains to be determined if our observation and resulting hypothesis that type I axon terminals, i.e. the cortical modulatory input reaching the pulvinar, increases along the cortical hierarchy can be generalized.

In summary, the present study shows that (1) as previously reported^17,65^, the primary visual cortex provides a modulatory input to first–order thalamic nuclei through type I terminals and conversely a much more important driving input to higher-order thalamic nuclei through type II terminals; (2) the PMLS cortex, located at a higher hierarchical level, provides mainly a modulatory input to first-order thalamic nuclei as shown by the very high proportion of type I terminals therein and both modulatory and driving signals through type I and II terminals in high-order thalamic nuclei^42^; (3) the AES cortex, which is considered as the highest hierarchical level among the three regions^24^, provides almost exclusively modulatory inputs to both first and high-order thalamic nuclei of the thalamus via type I terminals. Thus, there seems to be a general organization in which the primary visual cortex provides a prominent driving input to high-order thalamic nuclei while higher-order cortical areas exert predominantly a modulatory influence. These last signals can be part of mechanisms subtending attention–related gating at the thalamic level see^9,50,66^ and represent a demonstration of a top down regulation of cortical areas onto thalamic processing.

## Data Availability

The datasets generated during and/or analyzed during the current study are available from the corresponding author on reasonable request.
